# Takotsubu Cardiomyopathy Patient With Coronary Artery Disease: The Chicken or the Egg

**DOI:** 10.1002/ccr3.70028

**Published:** 2024-12-25

**Authors:** Chara Ude, Ayman Helal, Ibrahim Antoun

**Affiliations:** ^1^ Department of Cardiology Kettering General Hospital Kettering UK; ^2^ Department of Cardiovascular Sciences University of Leicester Leicester UK

**Keywords:** acute coronary syndrome, coronary artery disease, echocardiography, Takotsubu cardiomyopathy

## Abstract

This case emphasizes the rare occurrence of Takotsubo cardiomyopathy (TTC) in a patient with moderate coronary artery disease (CAD), highlighting the complexity of diagnosis and management. Clinicians should maintain a high index of suspicion for TTC in patients with CAD, especially when echocardiographic findings suggest apical ballooning. Balancing therapies for both conditions is essential.

## 1 Introduction

Takotsubo cardiomyopathy (TTC), commonly referred to as “broken heart syndrome,” is a transient form of cardiomyopathy that is often triggered by severe physical or emotional stress, resulting in a ballooning of the ventricular apex and cardiac dysfunction. It follows a similar pattern to acute coronary syndrome (ACS), with chest pain, electrocardiogram (ECG) abnormalities, and elevated cardiac biomarkers. The absence of coronary artery obstruction used to be the key distinguishing feature between TTC and ACS until recent studies began to disagree with this notion and reported that both could co‐exist and that there is a possibility that ACS could lead to TTC [1, 2], in which case there would be coronary artery disease but not significant enough to result in the severe obstruction that normally leads to ACS. The clinical overlap between TTC and ACS has remained a tough hurdle in making accurate diagnoses and providing adequate management.

We present a case of TTC in a patient with angiographic evidence of moderate CAD.

## 2 Case History and Examination

Our patient is a 44‐year‐old lady who was brought into the emergency room in our center on account of central crushing chest pain while in bed, which lasted for about 10–15 min, radiated to the jaw, forearm, and back, and was ranging between 7 and 10 on a 1–10 scale.

Her past medical history includes restless leg syndrome. She has been a smoker for the past 10 years. On that morning, she had a medical termination of pregnancy with misoprostol without significant bleeding upon presentation. Her vital signs were stable, but the physical examination was not significant.

## 3 Differential Diagnosis and Diagnostic Tests

**FIGURE 1:**
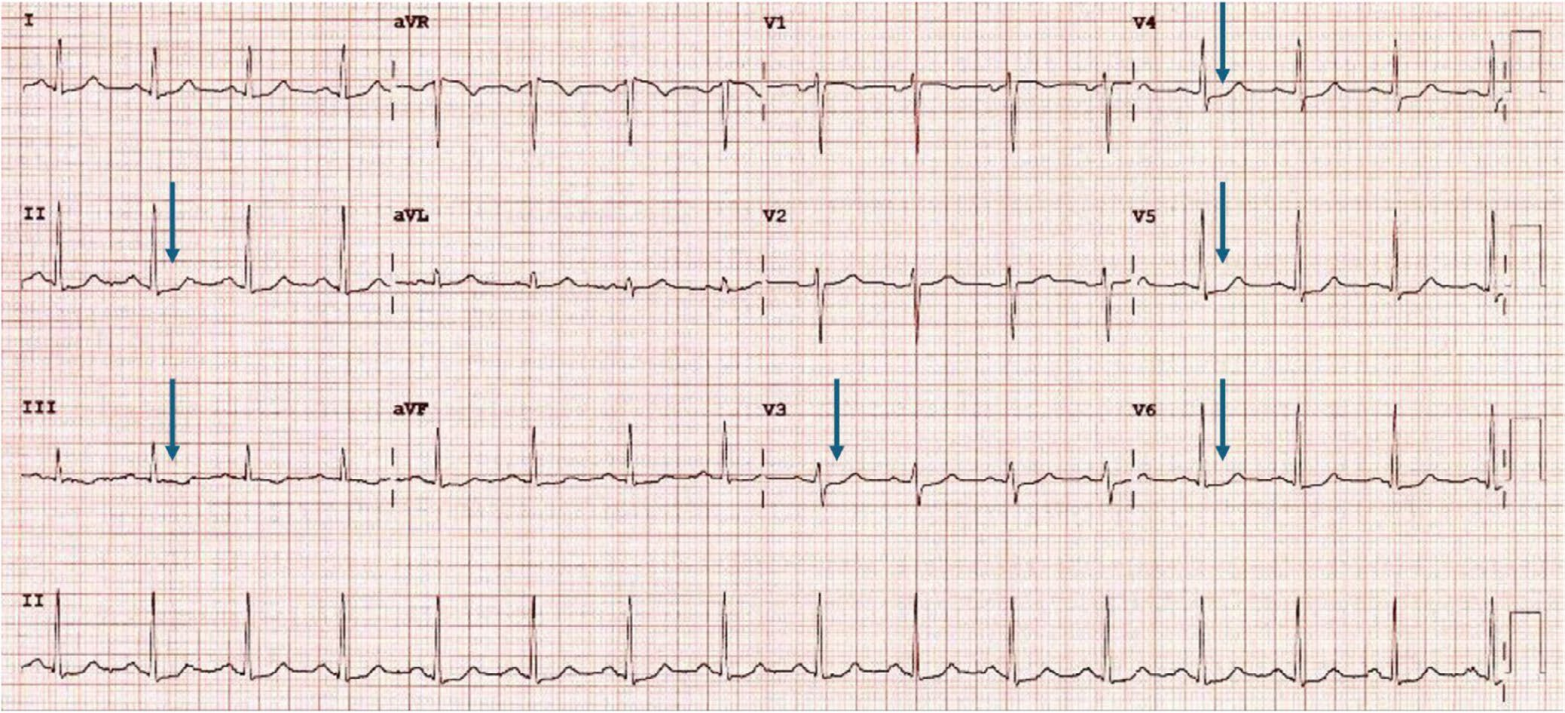
12‐leads electrocardiogram, demonstrating anterolateral and inferior ST depression (blue arrows).

The 12‐lead ECG showed sinus rhythm with ST depression (0.5–1 mm), noted in the anterolateral and inferior leads (Figure 1). Troponin levels rose from 34.9 to 124 ng/L (reference: 0–12 ng/L). Chest X‐ray (CXR) was normal.

**FIGURE 2:**
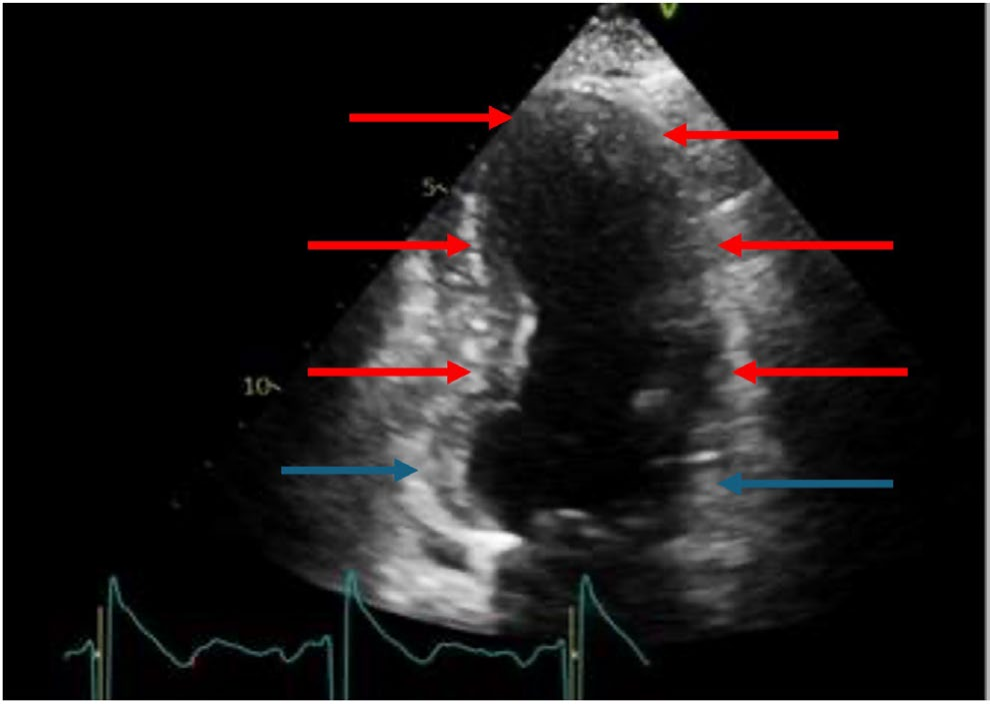
Apical two‐chamber echocardiographic view of the heart demonstrating preservation of the basal segments (blue arrows) while the rest of the myocardium is severely hypokinetic (red arrows). This is not in keeping with a single coronary artery territory and supports the diagnosis of Takotsubo cardiomyopathy, according to the Mayo Clinic diagnostic criteria.

Transthoracic echocardiography (TTE) demonstrated a normal‐sized left ventricle (LV) with impaired systolic function with an ejection fraction (EF) of 45%–50%. There was hypokinesia of the mid‐anterior and apical‐anterior LV wall segments, as well as akinesia of the mid‐anteroseptal, mid‐inferoseptal, apical septal, and apical inferior LV wall segments. The basal segments moved well, suggesting TTC (Figure 2). However, due to the smoking history, we decided to proceed with invasive coronary angiography (CAG). The differential diagnosis at this stage included ACS or TTC.

**FIGURE 3:**
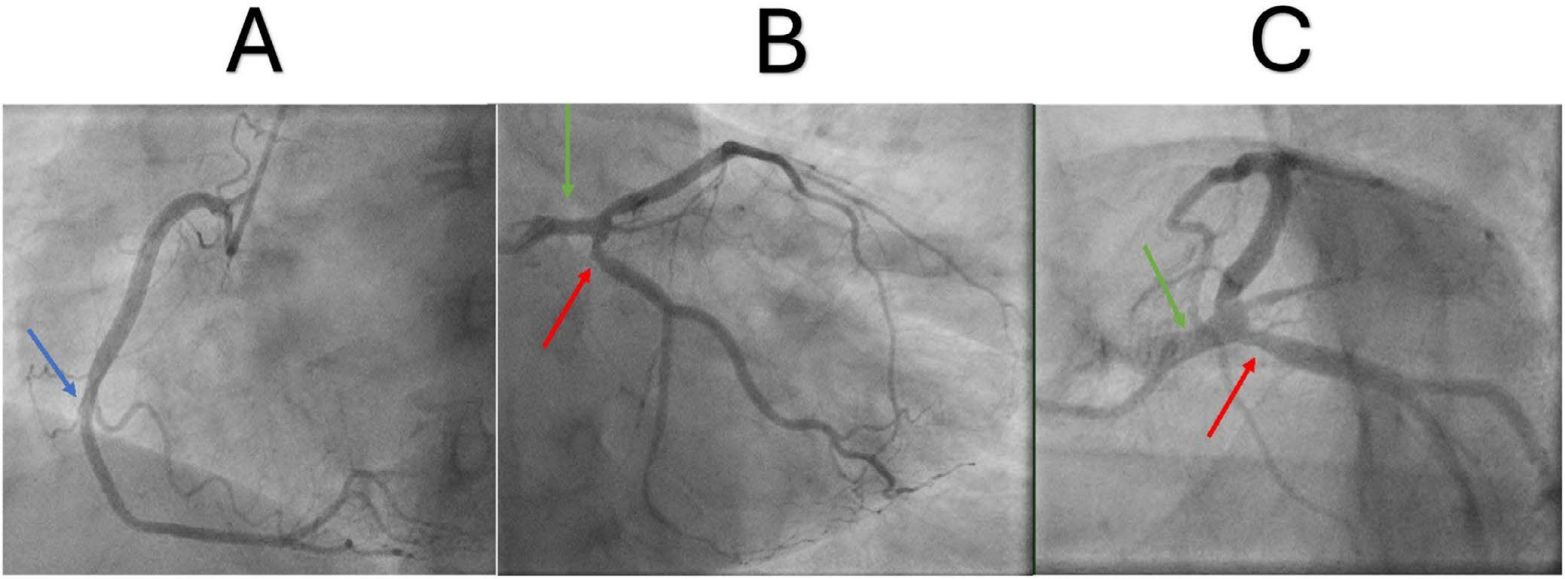
(A) Angiographic view of the right coronary demonstrating mild to moderate mid‐right coronary artery disease (blue arrow). (B, C) Angiographic views of the left coronary system demonstrating mild left main stem disease (green arrow) and moderate left circumflex disease (red arrow).

CAG did not reveal any significant vessel obstruction. However, mild left main stem (LMS) disease, moderate proximal left circumflex (LCX), and left anterior descending (LAD) disease were reported, with a conclusion of mild to moderate CAD (Figure 3).

After confirming mild to moderate CAD, the cardiology multidisciplinary team (MDT) discussed the case. The cardiologists debated whether CAD or TTC was the main issue. While moderate CAD was identified on CAG, the MDT thought process was that these findings did not correspond to the wall motion abnormalities noted on TTE. The distribution and reversible nature of the LV dysfunction on follow‐up TTE (showing normalization of wall motion abnormalities) further supported TTC as the primary diagnosis. Additionally, troponin elevation, though present, was mild compared to typical levels seen in ACS with obstructive CAD, which aligns with previously reported TTC cases where troponin levels are moderately elevated without severe coronary obstruction. These findings aligned with criteria outlined by the InterTAK Diagnostic Score, and Mayo Clinic guidelines for TTC were established [3], emphasizing transient wall motion abnormalities not confined to a single coronary artery distribution and lack of significant coronary artery obstruction. Therefore, the MDT came to the conclusion that TTC was the main diagnosis.

## 4 Results and Conclusion

The LV systolic function was treated with guidelines‐directed medical therapy (GDMT), including bisoprolol 2.5 mg once a day (OD) and ramipril 2.5 mg OD. Smoking cessation was advised, and the specialist team was involved.

A repeat TTE 6 weeks after the event showed complete resolution of the regional wall abnormalities and a return of LV systolic function to normal further supporting the diagnosis of TTC. The patient remained free of symptoms and managed to abstain from smoking.

## 5 Discussion

This case underscores the diagnostic challenge of differentiating TTC from CAD, mainly when there is an overlap between the two conditions. While echocardiographic findings were characteristic of TTC, mild to moderate CAD on CAG complicates the clinical picture. It raises critical considerations about whether CAD could have contributed to the presentation or if it was an incidental finding. Stress‐induced cardiomyopathy, commonly seen in postmenopausal women, may coexist with CAD, requiring a tailored approach to management that addresses both conditions. Long‐term prognosis favors recovery of ventricular function in TTC, but the presence of CAD necessitates ongoing cardiovascular risk assessment and management.

## 6 TTC

TTC, also known as stress‐induced cardiomyopathy or “broken heart syndrome,” is a transient condition characterized by LV systolic dysfunction, typically affecting postmenopausal women. However, younger women, as in this case, can also develop the syndrome, which presents similarly to ACS but without obstructive CAD in most cases. The hallmark of TTC is the apical ballooning of the LV, although other variants, such as mid‐ventricular or basal ballooning, are also recognized [4]. Diagnosis is often supported by imaging modalities such as TTE, CAG, and clinical history. Our patient's medical termination of pregnancy served as the stressor which provoked the TTC.

In this patient, TTE findings were consistent with the classic features of TTC, including regional wall motion abnormalities with apical ballooning. Despite moderate CAD, the distribution of the echocardiographic abnormalities did not correlate with the areas supplied by the affected coronary vessels, making TTC the more likely primary diagnosis. TTE was essential in the Mayo Clinic criteria, which included regional wall abnormalities beyond a single vessel territory [3]. Long‐term survival rates in TTC can be compared to the general population when no underlying comorbidities exist [1]. Most individuals stay symptom‐free after recovery. However, recurrences are uncommon yet possible [2]. A poor long‐term prognosis has been documented with co‐existing CAD [5]. As a result, these patients must be monitored after discharge and warned about circumstances that may raise the chance of recurrence. In the case of our patient, she was recommended to stop smoking. Untreated underlying causes, such as anxiety, might further increase the likelihood of recurrence. Although TTC is typically seen as a benign syndrome, the left ventricular dysfunction associated with it can result in consequences such as heart failure, intraventricular thrombus development, and systemic embolism. They should be monitored by echocardiography during the follow‐up.

## 7 CAD And TTC

A previous study proposed that TTC and CAD are not mutually exclusive disease entities. Excluding the diagnosis of TTC solely on an incidental finding of CAD may not be justified in all cases [6, 7]. Rather, a case‐by‐case decision process was indicated, such as in our case, which went to the cardiology MDT meeting, and the diagnosis of TTC was made based on the clinical history and TTE findings. A large cohort of 1016 TTC patients demonstrated that 64% of these patients had coexistent CAD, which was associated with worse clinical outcomes [5]. Moderate CAD complicates the clinical presentation in this case, as CAD could contribute to or exacerbate myocardial dysfunction. However, the distinct features of TTC, particularly the transient nature of the LV dysfunction and the pattern of wall motion abnormalities, helped us differentiate it from ischemic cardiomyopathy due to obstructive coronary lesions. As noted in clinical studies, patients with coexisting CAD and TTC may present with a more complex clinical course. However, their management does not differ significantly from those with TTC alone [1]. The patient's history of smoking represents an important factor in her cardiovascular risk profile, influencing both CAD and, potentially, TTC. Smoking is a well‐established risk factor for CAD, promoting atherosclerosis through oxidative stress, endothelial dysfunction, and inflammatory processes, which could contribute to the moderate CAD observed in CAG. However, smoking is also linked to heightened sympathetic nervous system activity, which may exacerbate stress responses and contribute to the onset of TTC.

Furthermore, a recent study correlated smoking with longer hospital stays in TTC patients [8]. Prior research indicates that nicotine and other components in cigarettes may elevate catecholamine levels [9], a known trigger for the adrenergic surge associated with TTC. In this context, it is possible that smoking increased her susceptibility to TTC by enhancing her physiological stress response to recent emotional and physical stressors, such as the medical termination of pregnancy. Consequently, addressing smoking cessation was particularly relevant in her management, aiming to reduce future risks associated with both CAD and the potential recurrence of TTC.

It is also essential to recognize that, in some instances, CAD may act as a precipitant for TTC, particularly in the case of coronary spasm or acute plaque rupture. However, in this patient, there was no evidence of acute plaque rupture or spasm during CAG, further supporting TTC as the primary condition. Nevertheless, the presence of CAD warrants long‐term management to reduce the risk of future ischemic events, even though the initial presentation was more consistent with TTC.

## 8 Clinical Implications and Management

The coexistence of TTC and moderate CAD poses diagnostic and management challenges. Treatment for TTC is primarily supportive, with most patients fully recovering left ventricular function within weeks to months. Long‐term outcomes are generally favorable, though recurrence rates have been reported in up to 10% of cases [10].

Early CAG was crucial in this patient to guide treatment and rule out obstructive CAD, especially when distinguishing between TTC and ACS was challenging. To avoid inappropriate treatment, awareness of the potential coexistence of TTC and CAD in some patients is essential. TTE is crucial in differentiating between TTC and ischemic heart disease when CAD is present but not severe enough to explain the observed myocardial dysfunction. This case contributes to the growing body of literature on TTC with CAD by illustrating how moderate CAD can coexist with TTC without significantly altering the diagnostic approach but requiring nuanced management to address both conditions effectively.

## Author Contributions

**Chara Ude:** writing – review and editing. **Ayman Helal:** writing – review and editing. **Ibrahim Antoun:** conceptualization, data curation, writing – original draft.

## Consent

The patient gave written informed consent to publish this report in accordance with the journal's patient consent policy.

## Conflicts of Interest

The authors declare no conflicts of interest.

## Data Availability

Data relating to this study are available upon reasonable request from the corresponding author.
